# Induced Electric Field Processing of Watermelon Juice: Effects on Microbial Inactivation, Physicochemical Stability, and Flavor Retention During Refrigerated Storage

**DOI:** 10.3390/foods15081426

**Published:** 2026-04-19

**Authors:** Yang Liu, Li-Li Li, Meng-Yao Fan, Zhi-Jing Ni, Run-Hui Ma, Zhao-Jun Wei, Kiran Thakur

**Affiliations:** 1 School of Biological Science and Engineering, North Minzu University, Yinchuan 750021, China; 17636362054@163.com (Y.L.); lililyplus@163.com (L.-L.L.); lovebear@vip.163.com (Z.-J.N.); zhantingbaiyang@163.com (R.-H.M.); zjwei@hfut.edu.cn (Z.-J.W.); 2 Ningxia Key Laboratory of Development and Utilization of Specialty Food Resources, Yinchuan 750021, China; 3 School of Food and Biological Engineering, Hefei University of Technology, Hefei 230009, China; 15555122657@163.com

**Keywords:** induced electric field, watermelon juice, shelf-life, quality attributes, volatile compounds

## Abstract

Watermelon juice is a nutritious yet highly perishable beverage. Conventional thermal pasteurization ensures safety but degrades heat-sensitive nutrients, color, and flavor. Induced electric field (IEF) is an emerging technology that inactivates microorganisms while better preserving quality. However, its effects on the comprehensive quality retention of watermelon juice during storage remain underexplored. This study investigated the efficacy of IEF treatment on the microbial inactivation and quality preservation of watermelon juice during 25 days of storage at 4 °C. Freshly extracted watermelon juice was subjected to low-temperature IEF at 65 °C (IEF1) for 101 s and 60 °C (IEF2) for 88 s, with conventional pasteurization (65 °C, 30 min) as a control. The results showed that no colonies were detected in the IEF2 group throughout the 25-day storage period. Both IEF treatment and pasteurization effectively inhibited juice acidification. Soluble solids content and electrical conductivity remained stable under refrigeration, and the IEF group showed slower and more controllable acidity on day 25. Notably, the IEF1 group retained the highest lycopene content at the end of storage, while the IEF2 group maintained the highest total phenolic content (TPC). Furthermore, IEF treatment effectively mitigated color deterioration and preserved carbohydrate stability during refrigeration. Flavor analysis revealed that the taste profile of the IEF2 group at the initial storage stage closely resembled that of fresh watermelon juice. Over the 25-day period, the relative content of key volatile compounds characteristic of fresh watermelon decreased by only 3.64% in the IEF2 group.

## 1. Introduction

Ningxia selenium-rich watermelons are a valuable agricultural product, prized for their content of carotenoids (including lycopene), vitamins, amino acids, and essential trace elements such as selenium. However, their high moisture content and heat-labile nature render them highly perishable, posing significant challenges for storage and transportation [1]. Processing fresh watermelon into juice is an effective strategy to reduce post-harvest loss, extend availability, and create a convenient, value-added product. While conventional thermal pasteurization is widely used to ensure microbial safety, its application often leads to the degradation of heat-sensitive nutrients, deterioration of fresh color, and loss of volatile aroma compounds, ultimately compromising the sensory profile and nutritional quality. Previous studies have reported that ultra-high-temperature (UHT) treatment can damage the heat-sensitive flavor, aroma, and nutritional compounds critical to watermelon juice quality [2,3].

To address these limitations, non-thermal and mild thermal technologies are increasingly being explored. Induced electric field (IEF) treatment has emerged as a high-potential, non-thermal technology for the processing of liquid beverages, offering a contactless alternative to conventional pulsed electric field methods. It utilizes a time-varying magnetic field to generate an induced electric current within the product, achieving microbial inactivation through a combination of Joule heating and postulated non-thermal effects (e.g., electroporation). For instance, Wu et al. [4] reported that IEF successfully maintained the microbiological safety and nutritional quality of apple juice at lower temperatures; He et al. [5] also found that IEF treatment extended the shelf life of kiwifruit juice. Recent research further supports IEF as a promising non-thermal pasteurization method, demonstrating its effectiveness in inactivating microorganisms and preserving quality attributes such as color and phenolic content in various fruit juices [5,6], including evidence of its success for shelf-life extension in bayberry juice [2,6]. Research indicates that IEF effectively ensures microbial safety, with studies on apple and orange juices reporting reductions in aerobic bacteria, yeasts, and molds by up to 5 log CFU/mL. Recent findings demonstrate superior retention of heat-sensitive nutrients including vitamin C, phenolics, and flavonoids while maintaining fresh-like color and flavor during storage. Furthermore, the application of electric fields to whole fruits can significantly enhance juice yield—in some cases by up to 28–40%—by inducing electroporation in cell membranes to facilitate mass transfer. Building on this foundation, IEF treatment has been shown to successfully maintain the physicochemical and microbiological quality of kiwifruit juice throughout storage [7,8]. Similarly, pulsed electric field (PEF) technology, a related electro-based method, has proven effective in preserving the overall quality of other fruit matrices, such as *Phyllanthus emblica* L. and its juice [9]. Meanwhile, UV-C is a feasible non-thermal alternative method for processing watermelon juice [10].

Despite the demonstrated efficacy of IEF in other fruit juices, its specific effects on watermelon juice, particularly on key flavor volatiles and phytochemicals during storage, have not been systematically investigated. Therefore, the primary objective of this study was to evaluate the effects of IEF treatment applied at different flow rates on microbial safety, physicochemical stability, and sensory quality of watermelon juice during 25 days of storage at 4 °C. Two IEF treatments were applied: IEF1 (65 °C, 101 s) and IEF2 (60 °C, 88 s). These parameters were selected to achieve effective microbial inactivation while maximizing the retention of characteristic flavor and nutrients. Following treatment, the juice was immediately analyzed for physicochemical and microbiological properties, including pH, soluble solids content, electrical conductivity, titratable acidity, total sugar, total phenolic content, and flavor profile. By providing a comprehensive comparison with traditional pasteurization, these findings offer novel insights and practical data to support the potential application of IEF technology in producing high-quality, shelf-stable watermelon juice.

## 2. Materials and Methods

### 2.1. Materials and Equipment

The plant material used was the Jin-Cheng No. 5 variety of selenium-enriched watermelon (also known as ‘Xisha melon’), obtained from the Hanjiaoshui Selenium-Enriched Watermelon Planting Base in Zhongwei City, Ningxia, China. Prior to processing, watermelons were carefully inspected; any specimens exhibiting physical damage, pest infestation, or inadequate ripeness were discarded to ensure consistent initial quality.

**Figure 1:**
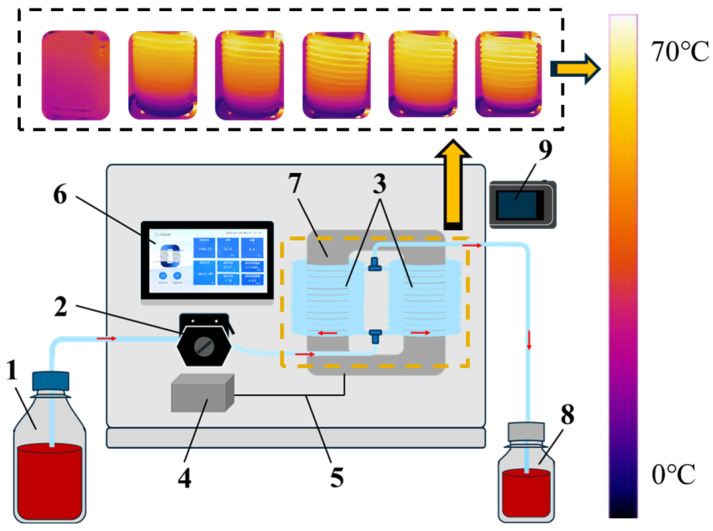
Schematic diagram of the IEF apparatus. 1. Sample vial. 2. Peristaltic pump. 3. Coil. 4. Power supply. 5. Excitation coil. 6. Control panel. 7. Magnetic core. 8. Sterile bottle. 9. Infrared thermometer.

The primary processing apparatus was an Induced Electric Field (IEF) unit (Model MP10, Biomag Innovations LLC, Sebastian, FL, USA). This system (Figure 1) generates a medium-frequency oscillating magnetic field via an excitation coil wound around a magnetic circuit. Juice was transported through this field by a peristaltic pump (Longer Pump, Baoding, China). Within the IEF treatment zone, electromagnetic coupling induces a current in the flowing juice, applying the desired electric field.

### 2.2. Treatment Procedure

Fresh watermelon juice was prepared immediately prior to each experiment. Medium-sized watermelons (cv. Jin-Cheng No. 5, approximately 5–6 kg each) were washed, peeled, and cut into small pieces (3–4 cm). The pieces were immediately juiced using a commercial juicer (Joyoung JYZ-V5 PLUS), and the freshly extracted juice was promptly transferred to sterile sample bottles. For each batch, two watermelons were used to ensure sufficient juice volume. All processing steps were carried out with minimal delay to prevent microbial growth and enzymatic activity.

Prior to each experiment, the processing environment was thoroughly sanitized. The treatment chamber and all work surfaces were exposed to ultraviolet (UV) light for 60 min, and the air was treated with an atomized sodium hypochlorite solution. The aseptic filling unit connected to the IEF outlet underwent a separate 30 min UV sterilization cycle. The internal IEF piping system was disinfected by pumping 2 L of 75% (*v*/*v*) ethanol through it, followed by a 5 min rinse with sterile distilled water.

Based on preliminary germicidal efficacy and quality experiments, fresh watermelon juice was treated at 6.5 L/h and 7.5 L/h volume flow rates, the terminal temperature was 65 °C (IEF1) and 60 °C (IEF2), respectively, and the residence time in the treatment circle was calculated to be about 101 s and 88 s, respectively. For comparative purposes, pasteurization was performed at 65 °C for 30 min. Following treatment, samples were immediately aliquoted into sterile bottles and stored at 4 °C for quality analysis at predetermined time points. The inlet juice temperature was maintained at 27 ± 1 °C. The outlet temperature, resulting from Joule heating, was recorded in real time using an infrared thermometer (Fluke, Everett, WA, USA). Treated juice was collected aseptically in pre-sterilized bottles. For comparison, a control batch was thermally pasteurized at 65 °C for 30 min in a water bath. All samples (Control, IEF1, IEF2, Pasteurized) were stored at 4 °C for 25 days. Analyses were performed on days 0, 5, 10, 15, 20, and 25. All the experiments, from raw material treatment to analysis, were performed in at least two independent juice batches.

### 2.3. Microbial Analysis

Total viable bacterial counts were determined using the standard plate count method [11] with modifications. Juice samples were serially diluted (10^−1^ to 10^−5^) in sterile physiological saline (0.85% *w*/*v* NaCl). One milliliter of appropriate dilutions was mixed with Plate Count Agar in Petri dishes. Plates were incubated at 36 ± 1 °C for 48 ± 2 h, after which colonies were counted. Results were expressed as log colony-forming units per milliliter (log CFU/mL).

### 2.4. Analysis of Physicochemical Properties

#### 2.4.1. pH, Soluble Solids, and Conductivity

The pH, total soluble solids (TSS, reported as °Brix), and electrical conductivity were measured according to a standard method [12]. A calibrated pH meter (PB-10, Sartorius, Göttingen, Germany) and a conductivity meter (FE30, Mettler Toledo, Greifensee, Switzerland) were used. TSS was determined using a digital refractometer (PAL-1, Atago, Japan). All instruments were calibrated prior to use, and samples were equilibrated to room temperature before measurement.

#### 2.4.2. Titratable Acidity

Titratable acidity was determined through potentiometric titration [13]. Briefly, 10 mL of juice was titrated with 0.02 M standardized sodium hydroxide (NaOH) solution to an endpoint of pH 8.1. TA was calculated as grams of citric acid equivalent per liter of juice (g CAE/L) using the formula

$$M=V\times \frac{C\times N\times K}{V_{1}}$$

where M is titratable acid content, V is the total volume of the sample (1000 mL), C is the volume of sodium hydroxide standard solution consumed (mL), N is the molar concentration of the standard solution of sodium hydroxide, V1 is the sample volume used for titration and 0.064 is the milliequivalent weight of citric acid (g/mmol).

#### 2.4.3. Total Phenolic Content

TPC was quantified using the Folin–Ciocalteu colorimetric method [14]. A 10 g aliquot of watermelon juice was transferred to a flask and extracted with methanol solution for 60 min. The mixture was then centrifuged at 2000 rpm for 15 min at 4 °C. An aliquot (0.2 mL) of the supernatant was transferred to a 25 mL test tube, followed by the addition of 1.0 mL of Folin–Ciocalteu reagent. The tube was vortexed thoroughly and allowed to stand in the dark for 5 min. Subsequently, 0.8 mL of 200 g/L Na_2_CO_3_ solution was added, mixed thoroughly, and the volume was adjusted to the mark with distilled water. The mixture was incubated at 30 °C for 30 min, and the absorbance was measured at 765 nm. Total phenolic content was determined by comparing the absorbance of each sample against a calibration curve constructed using gallic acid standards. Results were expressed as milligrams of gallic acid equivalents per 100 g of watermelon juice (mg GAE/100 g).

#### 2.4.4. Lycopene Content

Lycopene was extracted and quantified spectrophotometrically following a previously reported method [15]. An aliquot of watermelon juice (1 mL) was mixed with 25 mL of a solvent mixture comprising n-hexane, acetone, and anhydrous ethanol (2:1:1, *v*/*v*/*v*). The mixture was stirred magnetically at 4 °C for 15 min. Subsequently, 15 mL of distilled water was added, and stirring continued for an additional 5 min. The solution was allowed to stand for 15 min to separate into two distinct layers. The absorbance of the upper n-hexane layer was measured at 503 nm using a spectrophotometer. Lycopene concentration was determined by comparing the absorbance against a calibration curve constructed with lycopene standards.

#### 2.4.5. Color Measurement

Color parameters (L*, a*, b*) were measured using a colorimeter (CR-400, Konica Minolta, Tokyo, Japan) calibrated with a white standard tile. L* indicates lightness (0 = black, 100 = white), a* indicates red–green chromaticity (+a* = red, −a* = green), and b* indicates yellow–blue chromaticity (+b* = yellow, −b* = blue). The total color difference (ΔE) was calculated relative to the untreated day-0 sample:

$$\Delta E=\sqrt{{{(\Delta L}^{*})}^{2}+{{(\Delta a}^{*})}^{2}+{{(\Delta b}^{*})}^{2}}$$

#### 2.4.6. Total Sugar Content

Total sugar content was determined using the 3,5-dinitrosalicylic acid (DNS) method [16]. An aliquot of watermelon juice (1 mL) was mixed with 15 mL of distilled water and 10 mL of 6 M HCl, then hydrolyzed in a boiling water bath for 30 min. After cooling, one drop of phenolphthalein indicator was added, and the solution was titrated with 6 M NaOH until a faint pink color appeared. The neutralized solution was then diluted to 100 mL with distilled water and filtered. An aliquot (10 mL) of the filtrate was further diluted to 100 mL and mixed thoroughly.

From this diluted solution, 1 mL was transferred to a test tube and mixed with 1 mL of DNS reagent. The mixture was placed in a boiling water bath for 5 min, cooled to room temperature, and diluted to 10 mL with distilled water. Absorbance was measured at 540 nm using distilled water as a blank. Total sugar content was calculated by comparing the sample absorbance against a calibration curve constructed with glucose standards.

#### 2.4.7. Electronic Tongue Analysis

Taste profile analysis was performed using a taste-sensing system (TS-5000Z, Insent, Atsugi, Japan). The system comprised five sensor probes (C00, AE1, CA0, CT0, AAE) for basic tastes (umami, sour, bitter, astringency, aftertaste bitterness) and two reference electrodes. Sensors were activated and calibrated with standard solutions prior to sample analysis. Each sample measurement was performed in triplicate.

#### 2.4.8. GC-MS Analysis of Volatile Compounds

Volatile compounds were extracted through headspace solid-phase microextraction (HS-SPME) using a 50/30 μm DVB/CAR/PDMS fiber (Supelco, Bellefonte, PA, USA). An 8 mL juice sample in a 20 mL vial was equilibrated at 50 °C for 30 min with agitation. The pre-conditioned fiber was then exposed to the headspace for 20 min for adsorption.

Analysis was performed on a gas chromatograph–mass spectrometer (GC-MS QP2020 NX, Shimadzu, Kyoto, Japan) equipped with an Rxi-5ms capillary column (30 m × 0.25 mm, 0.25 μm film). The oven temperature program was 40 °C (hold 2 min), ramp at 4 °C/min to 80 °C, then at 2 °C/min to 140 °C (hold 3 min), and finally at 10 °C/min to 220 °C. Helium was the carrier gas at 1.0 mL/min. The injector and MS interface were at 250 °C. Mass spectra were acquired in electron ionization (EI) mode at 70 eV, scanning *m*/*z* 40–500.

Compound identification was achieved by comparing mass spectra with the NIST20 library and by matching calculated Kovats retention indices with literature values [17]. This analysis is a semi-quantitative method based on the percentage of total peak area.

### 2.5. Statistical Analysis

All experiments were performed in three biological replicates. Data were subjected to one-way analysis of variance (ANOVA) using SPSS software (v25.0, IBM, Armonk, NY, USA). Results are presented as mean ± standard deviation. The t-test was used to analyze the significance between the treated and control groups, *p* < 0.05 was considered statistically significant. Graphical representations were made using Origin software (v.2024, OriginLab, Northampton, MA, USA).

## 3. Results and Discussion

### 3.1. Temperature Rise Characteristics

An increase in input voltage leads to a significant enhancement in the amplitude of electron oscillations and induced current. The elevation in voltage promotes the conversion of more magnetic energy into thermal energy, thereby accelerating the temperature rise process. According to Joule’s law, the heat generated is also proportional to the square of the induced current density within the sample. It is noteworthy that IEF treatment enables uniform heat distribution, resulting in a steady and continuous temperature increase in the sample, which effectively enhances process efficiency [18].

**Figure 2:**
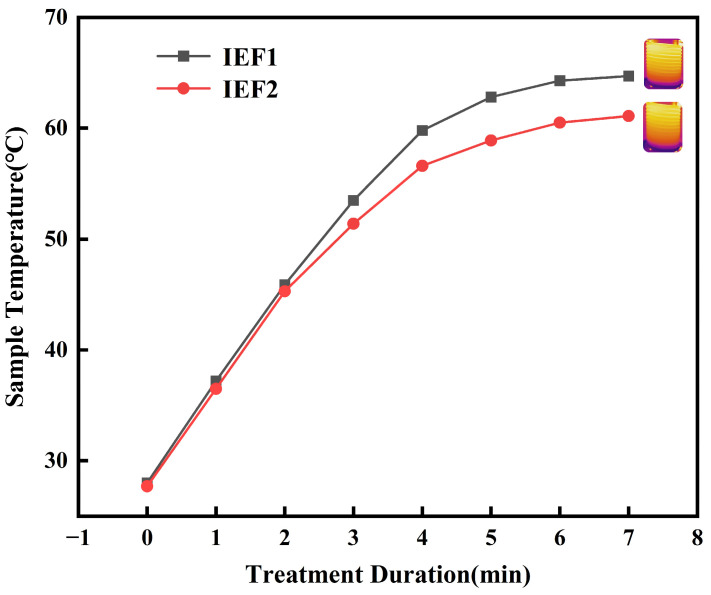
Temperature rise curves of the IEF1 and IEF2 treatment groups.

Figure 2 illustrates the temperature rise curves of the IEF1 and IEF2 treatment groups, showing that the final temperature of the IEF1 group is 65 °C, while that of the IEF2 group reaches 60 °C. This difference primarily stems from the variation in electric field strength [19,20]. Furthermore, the time required for the IEF treatment groups to reach the final temperature is influenced by multiple process parameters, including electric field strength, current magnitude, and fluid flow rate [21]. The IEF process ensures uniform temperature distribution in liquid food products under the oscillating magnetic field during the initial stage, achieving a linear temperature increase [19]. The small cross-sectional area of the pipeline may prevent localized overheating. This uniform heating process protects heat-sensitive components and achieves optimal inactivation [22,23].

### 3.2. Non-Thermal Effect

**Figure 3:**
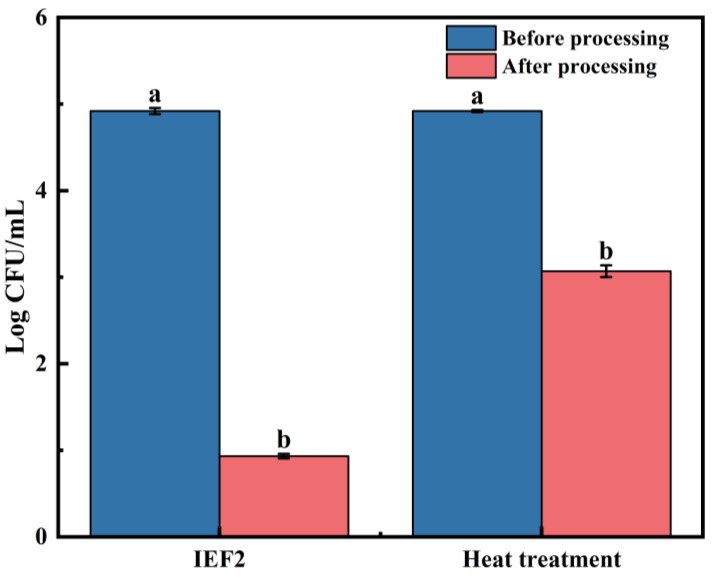
Effect of IEF2 and heat treatment under the same condition on the inactivation of watermelon juice. Different lowercase letters indicate significant differences in the same column (*p* < 0.05).

Figure 3 compares the microbial inactivation efficacy of IEF2 treatment (60 °C, 88 s) with conventional heat treatment at the same temperature and duration. Fresh watermelon juice had an initial microbial load of approximately 5 log CFU/mL. After IEF2 treatment, the viable count decreased to 0.93 log CFU/mL, whereas heat treatment alone only reduced it to 3.07 log CFU/mL. The superior inactivation by IEF suggests a non-thermal effect, likely due to electroporation of microbial cell membranes [24]. Additionally, the electric field may damage the bilayer structure of the cell membrane, further compromising microbial survival and proliferation [25].

### 3.3. Microbial Inactivation and Storage Stability

Microbiological safety is paramount for juice shelf-life. As shown in Table 1, the untreated control maintained a high microbial load (4.23–4.38 log CFU/mL) throughout storage. IEF treatment (IEF2, 7.5 L/h) achieved complete inactivation, with no detectable colonies over 25 days. IEF1 (6.5 L/h) was highly effective for the first 15 days, although minor microbial recovery (0.62–1.09 log CFU/mL) was observed thereafter. This suggests that IEF1 may have induced sub-lethal injury in a portion of the microbial population, allowing eventual repair during storage, a phenomenon noted in other electric field studies [26]. Notably, no colonies were detected in the IEF-treated group on day 15, while the pasteurized group showed approximately 0.56 log CFU/mL. By day 20, microbial counts in the IEF1 and pasteurized groups were reduced by 3.61 and 3.17 log CFU/mL, respectively, whereas the IEF2 group remained sterile throughout the entire 25-day storage period. Although pasteurization was initially effective, microbial regeneration was observed after day 15.

IEF2 treatment achieved a 4.23 log CFU/mL reduction immediately after processing and maintained microbial counts below the detection limit during 25 days of refrigerated storage at 4 °C. This sustained antimicrobial efficacy meets the core requirement for extended shelf life. The superior and sustained microbial inactivation by IEF2 highlights the critical role of treatment intensity (influenced by flow rate and residence time), aligning with findings that optimized IEF parameters ensure commercial sterility in acidic juices [5]. The greater inactivation efficacy of IEF compared to conventional heat treatment at the same temperature and duration (Figure 3) strongly suggests the contribution of non-thermal effects. IEF generates an induced electric current within the juice, which can compromise microbial cell membranes through electroporation [24,27]. This mechanism is supported by the observation that IEF2 achieved complete sterilization (no detectable colonies for 25 days), whereas heat treatment alone at 60 °C for 88 s reduced the microbial load only to 3.07 log CFU/mL. The electric field may also damage the bilayer structure of the cell membrane, further impairing microbial survival and proliferation [25]. However, the mild regrowth observed in the IEF1 group after day 15 indicates that sub-lethally injured cells may recover under refrigerated storage if treatment intensity is insufficient [26]. This parameter-dependent response highlights the importance of optimizing IEF parameters, particularly flow rate and residence time, to achieve commercial sterility. The baseline physicochemical properties of the juice were largely preserved by IEF treatment (Table 2). pH values showed a gradual decline in all samples during storage, a common trend due to organic acid production from microbial activity or residual enzyme action. However, the decline was significantly more severe in the untreated control. This decrease in pH value is probably due to the effective activation of pre-existing acidic metabolites in fruit juice, which significantly inhibits the growth and reproduction of microorganisms. The decrease in microbial activity, in turn, limits the continuous generation of acidic substances during storage, making the pH of the whole system maintain a dynamic balance at a relatively low level [28]. Both IEF and pasteurization effectively mitigated this acidification, maintaining pH above 5.9, which is crucial for flavor and stability. Total soluble solids fluctuated slightly across all treatment groups. These minor variations may be attributed to differences in the electric field parameters applied during processing, including voltage amplitude, treatment time, and outlet temperature. Importantly, the observed fluctuations confirm that IEF processing does not induce significant concentration or hydrolysis of soluble solids, a finding consistent with previous reports on IEF-treated bayberry juice [2].

**Figure 4:**
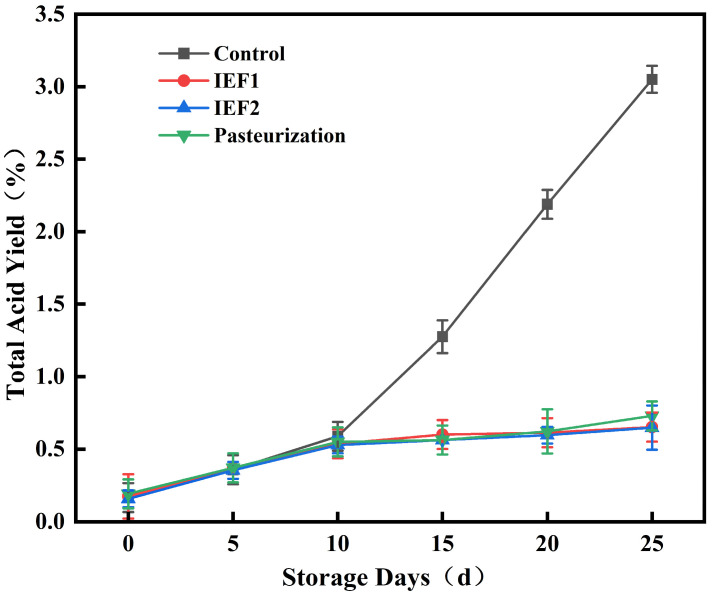
Changes in titratable acidity of watermelon juice during storage.

Conductivity is a key parameter reflecting the ionic strength of fruit juice. It directly influences the induced current high conductivity enhances the juice’s current transmission capacity, thereby reducing the impedance of the secondary coil. This reduction in impedance increases the output power of the secondary coil, improving the overall energy transfer efficiency of the magneto-electric coupling system. In this study, no significant changes in conductivity were observed across treatment groups, indicating stability in ionic composition during storage, consistent with previous reports [12]. Titratable acidity (Figure 4) increased in all samples over time, but the rise was most acute in the control, correlating with its microbial growth. The IEF-treated samples showed a slower, more controlled increase in acidity compared to the pasteurized sample by day 25. This suggests IEF may better inhibit the metabolic pathways leading to acid production, potentially through more effective enzyme inactivation [3].

### 3.4. Retention of Bioactive Compounds: Lycopene and Total Phenolics

**Figure 5:**
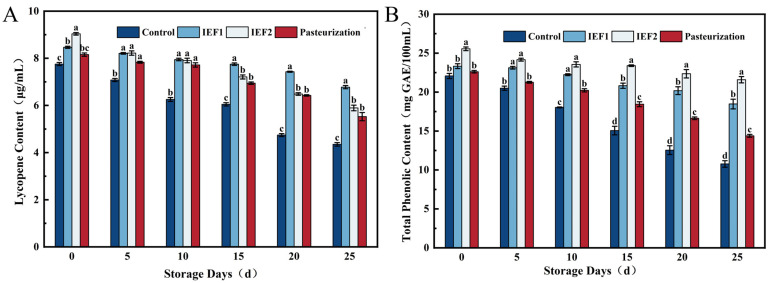
Changes in content of watermelon juice during storage: (**A**) Lycopene; (**B**) Total Phenolic. Different lowercase letters indicate significant differences in the same column (*p* < 0.05).

Preservation of health-promoting compounds is a key advantage of non-thermal technologies. Lycopene content declined during storage in all samples (Figure 5A), but the rate of degradation was treatment-dependent. Lycopene is a red carotenoid pigment highly susceptible to degradation under various environmental conditions due to its structure containing 11 conjugated double bonds [13]. The untreated control degraded the fastest. While pasteurization caused an initial thermal degradation, IEF1 (60 °C) showed the best protective effect, retaining the highest lycopene content on day 25. This superior retention can be attributed to the shorter thermal exposure and possible non-thermal effects of IEF that minimize oxidative and isomerization reactions detrimental to carotenoids [29]. Similar advantages in preserving heat-sensitive pigments have been reported for other fruit juices treated with electric fields [1].

Total phenolic content (TPC) also decreased over time but was better preserved by IEF treatments (Figure 5B). The initial higher TPC in treated samples (IEF and pasteurized) versus the control is likely due to heat-assisted extraction from cellular matrices [30]. Through storage, the IEF2 sample maintained the highest TPC, indicating that the specific IEF conditions (65 °C, 7.5 L/h) were most effective in inactivating oxidative enzymes like polyphenol oxidase (PPO) while minimizing thermal degradation of the phenolics themselves. This aligns with studies on IEF for kiwifruit juice, which reported enhanced phenolic stability [8], and underscores the technology’s capacity to preserve antioxidant potential [6]. Previous studies have reported that pulsed electric field (PEF) treatment can enhance the stability of phenolic compounds in orange juice, effectively preserving its bioactive components [29]. This protective effect is attributed to the complex interplay between processing parameters (e.g., electric field strength, treatment time) and subsequent storage conditions (e.g., temperature, duration). As phenolic substances are important antioxidants, improving their stability contributes to maintaining the overall health-promoting properties of the juice throughout its shelf life.

### 3.5. Color Stability

**Figure 6:**
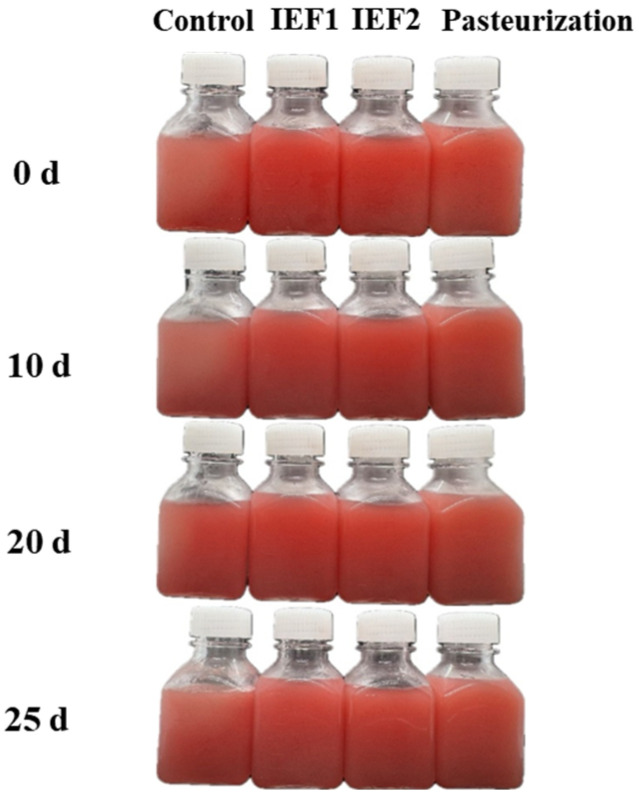
Visual appearance of watermelon juice samples during storage.

The visual appearance of watermelon juice samples throughout storage is presented in Figure 6. On day 0, all samples displayed a bright red hue, though the untreated control showed rapid stratification. Over 25 days, the control sample faded significantly, becoming lighter and cloudy, indicative of oxidation, sedimentation, and microbial spoilage. In contrast, IEF-treated juices maintained a more stable red-pink color, demonstrating better preservation of visual quality. Pasteurized juice exhibited a deeper, orange-red color that remained relatively stable, likely due to thermal concentration and Maillard browning.

**Figure 7:**
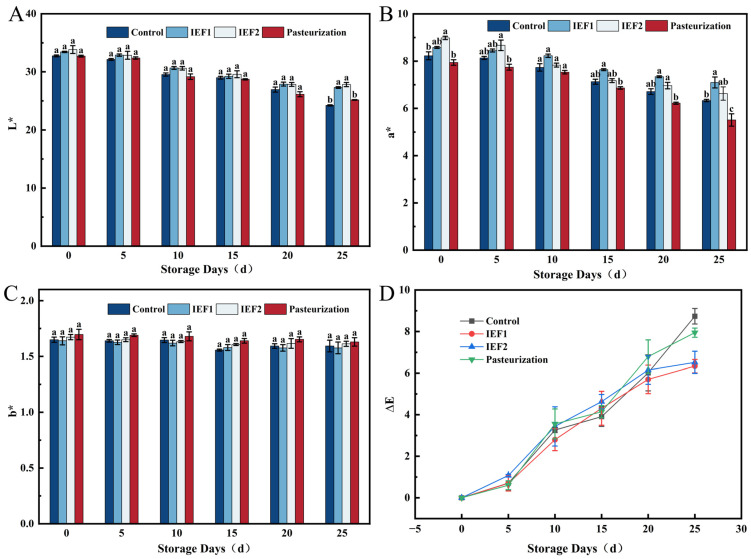
Color changes in watermelon juice during storage: (**A**) L*; (**B**) a*; (**C**) b*; (**D**) ∆E. Different lowercase letters indicate significant differences in the same column (*p* < 0.05).

IEF-treated juices maintained a more stable red-pink color over 25 days than the untreated control, which faded due to oxidation and microbial spoilage (Figure 6 and Figure 7). Pasteurized juice developed an orange-red tint from thermal concentration and Maillard browning. The superior color retention by IEF is attributed to its milder thermal impact, better lycopene preservation, and inhibition of enzymatic browning, consistent with reports on IEF-treated berry juices [2,6].

### 3.6. Total Sugar Content

**Figure 8:**
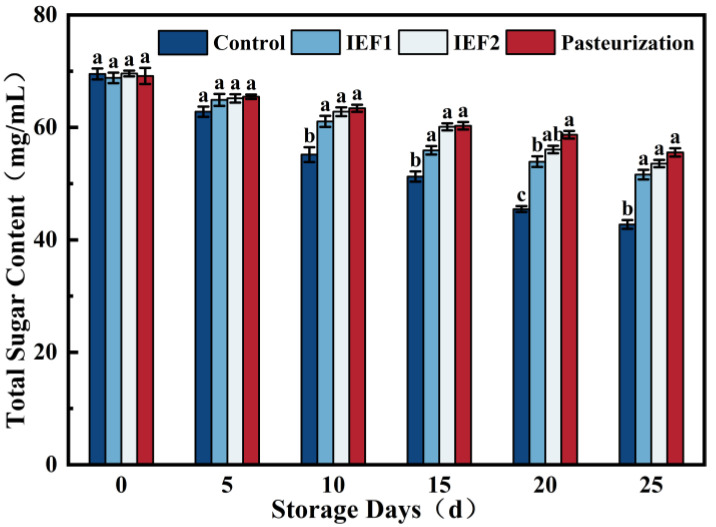
Changes in total sugar content of watermelon juice during storage. Different lowercase letters indicate significant differences in the same column (*p* < 0.05).

Total sugar content gradually decreased in all samples during storage (Figure 8), likely due to microbial and residual enzymatic activity. The IEF-treated and pasteurized samples showed similar, more stable sugar profiles compared to the sharp decline in the control. The slightly higher sugar concentration in the pasteurized sample at later time points may be attributed to slight water evaporation during the prolonged thermal hold [2].

### 3.7. Flavor Profile: Electronic Tongue and Volatile Aroma Compounds

**Figure 9:**
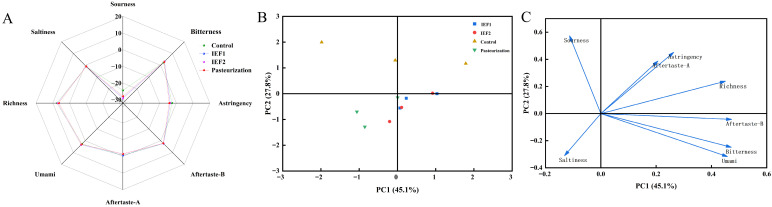
Analysis of electronic tongue at the early stage of cold storage: (**A**) radar chart; (**B**) score plot; (**C**) loading plot. Different lowercase letters indicate significant differences in the same column (*p* < 0.05).

The electronic tongue analysis (Figure 9) revealed that IEF-treated juice, particularly IEF1, had a taste profile closer to the original, untreated juice than the pasteurized sample profile. Pasteurization resulted in detectable deviations, particularly in richness and sourness. Principal Component Analysis (PCA) clearly separated the samples based on processing, with IEF samples clustering closer to the control, indicating less alteration of the overall taste balance.

**Figure 10:**
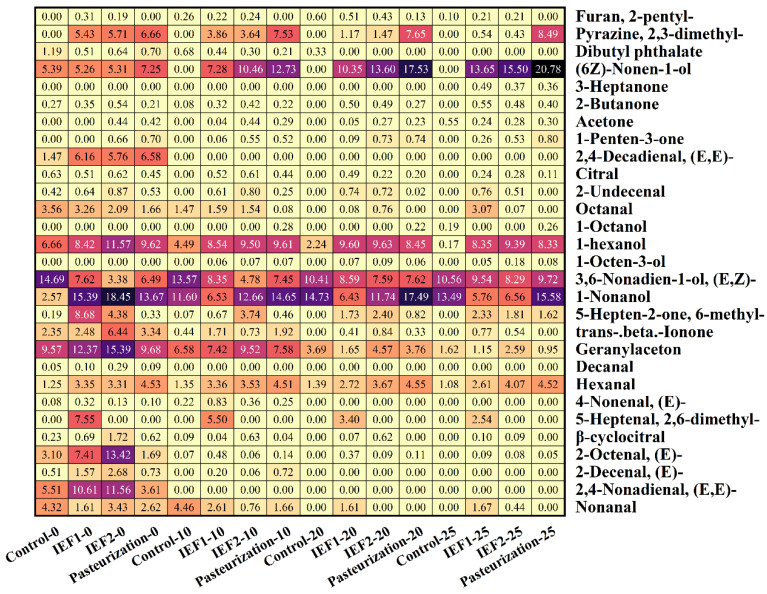
Relative content of volatile compounds in watermelon juice during storage.

The analysis of volatile compounds in this study used the percentage of peak area for semi-quantitative comparison. GC-MS analysis of volatile compounds provided a detailed picture of aroma preservation (Table 3, Figure 10). The fresh, green, and fruity notes of watermelon juice, contributed by aldehydes (nonanal, hexanal) and alcohols, were better retained in IEF-treated samples. Pasteurization led to a greater loss of these desirable fresh-top notes and a higher relative concentration of off-flavors or degradation compounds by day 25. The relative content of key fresh-character volatiles decreased by only 3.64% in IEF2 over 25 days, compared to a 14.3% loss in pasteurized juice. This demonstrates IEF’s significant advantage in preserving the delicate and heat-labile aroma profile of watermelon juice, a finding echoed in research on PEF-treated fruits [9] and other IEF-processed juices [5].

Nonanal, (E, E)-2, 4-nonadienal, 2-decenal, citral, β-cyclodecal, 2, 6-dimethyl-5-heptenal, (E)-4-nonenal, octyl aldehyde, geranyl, cis-β-ionic ketone, acetone, 2-butanone, 1-nonanol, 3, 6-nonadien-1-ol, 1-hexanol, cis-6-nonen-1-ol, and dibutyl phthalate are the main volatiles of watermelon juice. On day 25, nonanal was detected only in IEF1 (1.67%) and IEF2 (0.44%), whereas it was absent in pasteurized juice. Similarly, β-cyclocitral was detected only in IEF-treated samples (0.24%), and citral was better preserved in IEF2 (0.28%) than in pasteurized juice (0.11%). The relative content of geranylacetone gradually decreased during cold storage, but the IEF2 group retained 2.59% on day 25. Notably, cis-β-Ionone was detected only in the IEF group, and both were related to pulp color and sweetness, giving a sense of recall hierarchy. Furthermore, the relative content of cis-6-nonen-1-ol increased after IEF treatment, suggesting that IEF may enhance the extraction or formation of certain volatile compounds.

The compound 6-methyl-5-hepten-2-one, which is associated with odor, was partially removed during processing. This may be explained by volatilization or aroma dragging during treatment. During refrigerated storage, its sweetness and citrus-like notes may decrease in parallel with its relative content [31].

## 4. Conclusions

This study demonstrates that induced electric field (IEF) treatment effectively inactivated microorganisms in watermelon juice while preserving its physicochemical, nutritional, and sensory properties compared to conventional pasteurization. IEF2 (60 °C, 88 s) provided superior microbial control, maintaining sterility throughout 25 days of refrigerated storage. IEF1 (65 °C, 101 s) was more effective at retaining lycopene and color stability. Both IEF treatments outperformed pasteurization in maintaining phenolic content and fresh-like volatile compounds. These findings support IEF as a viable non-thermal technology for watermelon juice processing, with potential for scaling through parallel flow systems. IEF offers practical advantages over thermal pasteurization (contactless operation, no prolonged heat exposure, and cold-chain compatibility), but limitations include laboratory-scale validation, a 25-day storage duration, lack of sensory confirmation, and unknown economic feasibility. Future studies should address scale-up, longer storage trials, sensory evaluation, and cost analysis.

## Figures and Tables

**Table 1:** Changes in colony counts of watermelon juice during storage.

**Total** **viable counts**	**Storage (d)**	**Control**	**IEF1**	**IEF2**	**Pasteurization**
	0	4.23 ± 0.02 ^a^	Nd	Nd	Nd
	5	4.25 ± 0.01 ^a^	Nd	Nd	Nd
	10	4.28 ± 0.03 ^a^	Nd	Nd	Nd
	15	4.32 ± 0.01 ^a^	Nd	Nd	0.56 ± 0.08 ^b^
	20	4.35 ± 0.01 ^a^	0.62 ± 0.05 ^c^	Nd	1.06 ± 0.03 ^b^
	25	4.38 ± 0.02 ^a^	1.09 ± 0.02 ^c^	Nd	1.84 ± 0.07 ^b^

Different lowercase letters indicate that there are significant differences between different treatment groups (*p* < 0.05). Nd for not detected.

**Table 2:** Physicochemical characteristics of watermelon juice during storage.

Parameters	Storage (d)	Control	IEF1	IEF2	Pasteurization
pH	0	6.21 ± 0.02 ^a^	6.2 ± 0.01 ^ab^	6.19 ± 0.01 ^ab^	6.18 ± 0.01 ^b^
	5	6.15 ± 0.02 ^a^	6.14 ± 0.03 ^a^	6.15 ± 0.02 ^a^	6.12 ± 0.01 ^a^
	10	5.92 ± 0.01 ^c^	6.06 ± 0.01 ^a^	6.07 ± 0.03 ^a^	6.02 ± 0.01 ^b^
	15	5.73 ± 0.01 ^c^	6.04 ± 0.01 ^a^	6.05 ± 0.02 ^a^	5.97 ± 0.02 ^b^
	20	5.63 ± 0.02 ^c^	6.01 ± 0.02 ^a^	6.02 ± 0.01 ^a^	5.93 ± 0.02 ^b^
	25	5.45 ± 0.04 ^c^	5.93 ± 0.02 ^ab^	5.96 ± 0.02 ^a^	5.89 ± 0.01 ^b^
TSS (°Brix)	0	10.3 ± 0.26 ^b^	10.5 ± 0.26 ^b^	11.1 ± 0.15 ^a^	10.1 ± 0.25 ^b^
	5	10.3 ± 0.26 ^a^	10.3 ± 0.10 ^a^	10.2 ± 0.21 ^a^	10.0 ± 0.1 ^a^
	10	10.3 ± 0.40 ^a^	10.2 ± 0.25 ^a^	10.6 ± 0.59 ^a^	10.1 ± 0.25 ^a^
	15	10.3 ± 0.21 ^a^	10.1 ± 0.15 ^a^	10.2 ± 0.10 ^a^	9.7 ± 0.06 ^b^
	20	9.1 ± 1.01 ^b^	10.7 ± 0.20 ^a^	10.9 ± 0.17 ^a^	9.9 ± 0.15 ^ab^
	25	9.3 ± 0.44 ^c^	10.8 ± 0.1 ^a^	10.8 ± 0.25 ^a^	10.2 ± 0.12 ^b^
Conductivity (µS/cm)	0	3.22 ± 0.01 ^a^	3.23 ± 0.00 ^a^	3.22 ± 0.01 ^a^	3.22 ± 0.01 ^a^
	5	3.22 ± 0.01 ^a^	3.22 ± 0.02 ^a^	3.22 ± 0.02 ^a^	3.22 ± 0.01 ^a^
	10	3.23 ± 0.00 ^a^	3.22 ± 0.01 ^a^	3.23 ± 0.02 ^a^	3.23 ± 0.01 ^a^
	15	3.22 ± 0.01 ^a^	3.22 ± 0.00 ^a^	3.23 ± 0.01 ^a^	3.22 ± 0.01 ^a^
	20	3.22 ± 0.00 ^a^	3.22 ± 0.01 ^a^	3.23 ± 0.00 ^a^	3.23 ± 0.01 ^a^
	25	3.22 ± 0.00 ^a^	3.22 ± 0.02 ^a^	3.22 ± 0.02 ^a^	3.23 ± 0.01 ^a^

Different lowercase letters indicate that there are significant differences between different treatment groups (*p* < 0.05).

**Table 3:** Volatile compounds identified in watermelon juice during storage.

Category	Compound	CAS Number	Odor Description
Aldehydes	Nonanal	124-19-6	Fatty, fresh scent
	(E,E)-2,4-Nonadienal	5910-87-2	Fatty, fresh scent
	(E)-2-Decenal	3913-81-3	Soapy, mechanical odor
	2-Undecenal	2463-77-6	Sweet scent
	(E)-2-Octenal	2548-87-0	Fatty, nutty
	Citral	5392-40-5	Lemon scent
	β-Cyclocitral	432-25-7	Minty
	(E,E)-2,4-Decadienal	25152-84-5	Fried, fatty odor
	2,6-Dimethyl-5-heptenal	106-72-9	Fruity fragrance
	(E)-4-Nonenal	2277-16-9	Fruity
	Hexanal	66-25-1	Grassy
	Decanal	112-31-2	Soapy, pungent
	Octanal	124-13-0	Fresh, fatty
Ketones	Geranylacetone	3796-70-1	Floral, fresh, sweet
	cis-β-Ionone	79-77-6	Floral, oat-like
	6-Methyl-5-hepten-2-one	110-93-0	Mushroom, rubber
	1-Penten-3-one	1629-58-9	Garlic, onion
	3-Octanone	106-68-3	Herbal, fatty
	Acetone	67-64-1	Sweet, rubber
	2-Butanone	78-93-3	Floral
Alcohols	1-Nonanol	143-08-8	Fatty, fresh
	3,6-Nonadien-1-ol	56805-23-3	Fresh, cucumber-like
	1-Octen-3-ol	3391-86-4	Mushroom
	1-Hexanol	111-27-3	Floral, fresh
	1-Octanol	111-87-5	Metallic, burnt
	cis-6-Nonen-1-ol	35854-86-5	Fresh, cucumber-like
Esters	Dibutyl phthalate	84-74-2	Aromatic
Others	2,3-Dimethylpyrazine	5910-89-4	Nutty, caramel
	2-Pentylfuran	3777-69-3	Caramel, musty

The scent description originates from: http://www.flavornet.org.

## Data Availability

The original contributions presented in this study are included in the article. Further inquiries can be directed to the corresponding author.
